# The roles of RNA in DNA double-strand break repair

**DOI:** 10.1038/s41416-019-0624-1

**Published:** 2020-01-02

**Authors:** Aldo S. Bader, Ben R. Hawley, Ania Wilczynska, Martin Bushell

**Affiliations:** 1 Cancer Research UK Beatson Institute, Glasgow, G61 1BD UK; 2 Department of Pharmacology, Weill Cornell Medicine, Cornell University, New York, NY 10065 USA; 3 Institute of Cancer Sciences, University of Glasgow, Glasgow, G61 1QH UK

**Keywords:** Double-strand DNA breaks, Data integration, DNA damage response, Cancer genomics, RNA metabolism

## Abstract

Effective DNA repair is essential for cell survival: a failure to correctly repair damage leads to the accumulation of mutations and is the driving force for carcinogenesis. Multiple pathways have evolved to protect against both intrinsic and extrinsic genotoxic events, and recent developments have highlighted an unforeseen critical role for RNA in ensuring genome stability. It is currently unclear exactly how RNA molecules participate in the repair pathways, although many models have been proposed and it is possible that RNA acts in diverse ways to facilitate DNA repair. A number of well-documented DNA repair factors have been described to have RNA-binding capacities and, moreover, screens investigating DNA-damage repair mechanisms have identified RNA-binding proteins as a major group of novel factors involved in DNA repair. In this review, we integrate some of these datasets to identify commonalities that might highlight novel and interesting factors for future investigations. This emerging role for RNA opens up a new dimension in the field of DNA repair; we discuss its impact on our current understanding of DNA repair processes and consider how it might influence cancer progression.

## Background

Genomic stability is ensured by the DNA-damage response (DDR), a network of pathways that functions to sense, repair and initiate cellular responses to any genotoxic damage incurred. DNA damage occurs regularly in response to both exogenous and endogenous queues, and a lack of, or ineffective, DNA repair might lead to genomic alterations – point mutations, insertions, deletions, expansions/contractions of repetitive sequences and translocations across the genome – which can ultimately result in cell-cycle arrest and eventual cell death if they accumulate.^1–3^ In addition, a failure to maintain genome fidelity in response to DNA damage is a significant contributor to carcinogenesis and underlies the capacity of cancer cells to adapt under selection pressures.^4–6^

The DDR comprises multiple pathways, each of which recognises and resolves specific types of DNA damage. Such damage includes large nucleotide adducts, which are resolved by nucleotide excision repair; small lesions, resolved by base excision repair;^7,8^ and double-strand breaks (DSBs), resolved by pathways such as non-homologous end-joining (NHEJ) and homologous recombination (HR).^1,9,10^ The various pathways involved in the DDR have been well studied at the molecular level, resulting in comprehensive mechanistic understanding behind their modes of action. However, a growing body of evidence suggests that RNA plays a significant role in the repair of DNA damage through currently unresolved mechanisms.^11–18^ The emerging role of transcription, RNA-interacting proteins, RNA-processing enzymes, and RNA itself, in the repair of DNA damage is becoming increasingly apparent,^19–22^ and understanding the contribution that RNA makes to the DDR will provide a new level of insight into genome maintenance. The concept of RNA-dependent DNA repair (RDDR) has the potential to alter our understanding of, and prospects for, cancer research and therapy, especially considering the recent emergence of RNA therapies.^23,24^ The development of RNA therapeutics is providing a series of novel treatment targets and strategies for a variety of conditions, including cancer.^25–28^ RDDR has the potential to allow these therapies to augment DNA repair through manipulation of the RNA substrates it relies on, possibly providing novel, sequence-dependent treatment targets. Here we discuss the new, pivotal research in this area and attempt to shed light on the possible mechanisms for the involvement of RNA in the DDR, with a focus on DSB repair due to its clinical significance and the breadth of data available.

## The DNA double-stranded break response: an overview

**Fig. 1:**
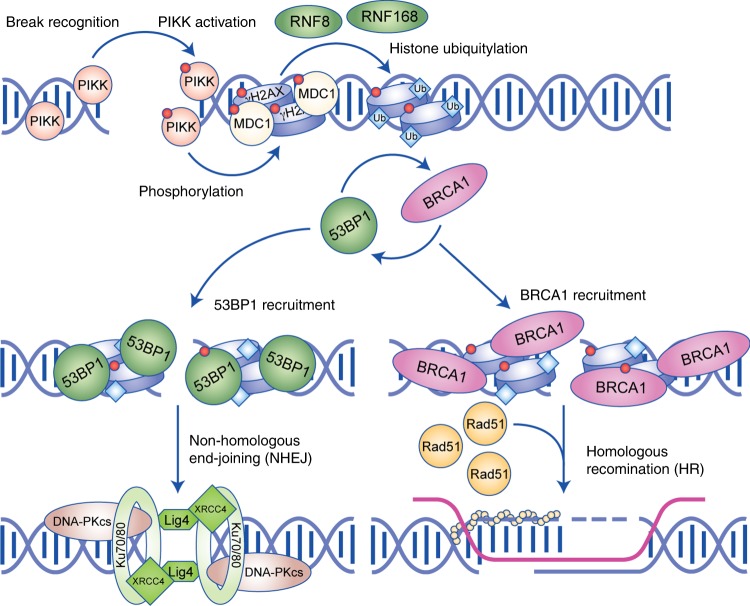
Schematic diagram of the double-stranded break (DSB) repair cascade. Initial recognition of the break recruits the phosphatidylinositol 3-kinase-like kinases (PIKKs), resulting in its activation by autophosphorylation. Activated PIKKs then phosphorylates early participants of the cascade, H2AX and MDC1. The ubiquitin ligase RNF8 recognises and is recruited to phosphorylated MDC1 at the break site, initiating the ubiquitylation of histone H1, which subsequently recruits RNF168 to ubiquitylate H2A. The modified chromatin acts as a marker for the competitive recruitment of either 53BP1 or BRCA1, which facilitate either non-homologous end-joining (NHEJ) or homologous recombination (HR), respectively. NHEJ repair is dependent on the recruitment of the DNA–PK complex (Ku70–80 bound to DNA–PKcs) and end-processing factors such as XRCC4 and Lig4. HR is dependent on resection of the broken DNA to produce a single-stranded DNA overhang that gets coated in a RAD51 filament and invades the sister-chromatid to facilitate templated repair.

DSBs are the most genotoxic of all forms of DNA damage and have a high propensity for resulting in insertions, deletions, translocations and even copy number variations in the genome, making their efficient repair critical for cell survival and the suppression of carcinogenesis.^2,3,6,10^ NHEJ and HR are the two major DSB repair pathways, with NHEJ being a fast, non-specific ligation and HR being a slower, higher fidelity pathway.^10^ HR utilises the sister-chromatid as a template for repair and therefore primarily occurs in S- and G2-phase of the cell cycle when the sister-chromatid is available, whereas NHEJ occurs throughout the cell cycle. Both pathways utilise the same signalling propagation cascade to drive the cellular responses to the DSB, initiated by activation of the phosphatidylinositol 3-kinase (PI3K)-like kinases (PIKKs): ataxia telangiectasia mutated (ATM), ataxia telangiectasia and rad3-related (ATR) and DNA-dependent protein kinase (DNA-PK) in response to induction of a break (Fig. 1).^29,30^ The PIKKs carry out key phosphorylation events, including the phosphorylation of histone H2AX (γH2AX), which acts as a central marker in DDR signalling. PIKK-dependent phosphorylation of Chk1/2 and p53 also mediates overall cellular responses, leading to cell-cycle arrest and the upregulation of repair factor gene expression.^31–33^ The recruitment of MDC1, another PIKK substrate, to γH2AX facilitates the recruitment of the E3 ubiquitin ligases, such as RNF8 and RNF168, which then mediate polyubiquitylation of the H1-linker histone and H2A.^34–36^ P53-binding protein 1 (53BP1) is then recruited to ubiquitin modifications on H2A. The retention of 53BP1 at the break then facilitates NHEJ; however, 53BP1 can be removed from the break site by BRCA1–CtIP binding to promote HR (Fig. 1).^37–39^

NHEJ occurs via binding of the Ku70–80 heterodimer, which sequesters the broken DNA ends and recruits factors, such as DNA-dependent protein kinase catalytic subunit (DNA-PKcs), XRCC4, Ligase IV and Artemis, that process and ligate the ends to resolve the break.^40–42^ DSB end-processing during NHEJ involves multiple processes, such as phosphorylation and short-range resection of broken DNA in the 5′–3′direction to provide a single-stranded DNA (ssDNA) overhang. NHEJ provides a mechanism that rapidly repairs DSBs and can occur during any phase of the cell cycle. However, the lack of any fidelity checks means that NHEJ is prone to the insertion or deletion of bases – indels – and translocations.^2,10^ By contrast, HR requires the long-range resection ssDNA overhangs up to kilobases from the break. This ssDNA gets coated in a RAD51 protein filament to allow the invasion of the ssDNA overhang into the double-stranded sister-chromatid (Fig. 1).^43^ The sister-chromatid is used as a template to re-polymerise the resected strand ready for ligation, thereby preventing any loss of information or illegitimate ligation of different DSB ends. The balance between the fast NHEJ and the high-fidelity HR responses to DSBs creates a symbiotic relationship between the two pathways, which provides robust genomic repair.

Defects in components of the DDR pathway can lead to the rapid accumulation of genomic instability, consequently promoting cancer development and progression; mutations in genes that encode DSB repair factors are therefore common in cancer cells.^3,5,6^ This is highlighted by the association of cancer-prone syndromes with the inheritance of defective DSB repair genes, such as BRCA1/2 and ATM/ATR.^44–47^ However, DDR pathway components can also be therapeutically targeted in an attempt to induce damage and death in cancer cells via chemotherapeutics that induce DSBs, such as cisplatin,^48,49^ as well as targeted therapeutics such as Olaparib.^50,51^ Olaparib inhibits the poly-ADP ribose polymerase (PARP) family of proteins, including the DNA repair enzyme, PARP1, and is used to treat patients with BRCA1/2 mutations whose cells are more reliant on PARP1 for DNA repair.^50,51^

## RNA-interacting enzymes in the repair of DNA damage

Our understanding of the contribution of RNA to the DDR and genome maintenance is expanding rapidly with well-known RNA-binding/processing enzymes being identified as novel players in these processes.^11,13,16,52,53^ Canonical components of microRNA (miRNA) biogenesis, splicing and transcriptional regulation machineries have been shown to have a variety of critical roles in the repair of DNA damage.^11,13,15,17,18,52,54–58^ In addition, a number of well-studied DNA repair proteins have been identified to have RNA-binding motifs and to interact with RNA-processing enzymes in a manner that facilitates DNA repair.^55,58,59^

### Components of miRNA biogenesis, splicing and transcriptional regulation in the DDR

The RNA endonucleases Drosha and DICER are core components of the miRNA biogenesis machinery, but have been shown to be required for propagation of the DDR.^12,13,17^ Depletion of Drosha and DICER results in deficient recruitment of repair factors to the damaged site and reporter assays show a significant reduction in both HR and NHEJ repair efficiency, comparable with that seen after BRCA1 and 53BP1 depletion, respectively.^12,17^ This role in the DDR is distinct from Drosha and Dicer’s roles in miRNA biogenesis, as the depletion of other factors in this biogenic pathway does not significantly hinder DNA repair.^13,17^ Immunofluorescence studies found that Drosha acts at the DDR ubiquitin cascade, as Drosha depletion significantly hindered the recruitment of factors from RNF168 onwards and impaired damage-induced ubiquitylation.^17^ In addition, Drosha has been shown to localise to break sites, and to be required for processing of RNA in response to DSBs to generate DNA*–*RNA hybrids around the damaged loci.^12–14,17^

RNA-splicing factors such as NONO and THRAP3^55,56^ have also been implicated in DNA repair and might represent a sub-family of RNA-processing proteins involved in the DDR.^55,56,60^ In addition, research from the Farnebo lab has identified WRAP53β, the small Cajal body-specific RNA (scaRNA)-regulating protein, as being essential for DDR signalling.^60,61^ Similar to the phenotype shown following Drosha depletion, recruitment of repair factors from RNF8 in the DDR ubiquitin cascade onwards is abrogated following WRAP53β depletion, while upstream factor recruitment remains unperturbed.^17,61^ With growing evidence that the DDR ubiquitin cascade is a complex and critical signalling step of histone modifications,^34,62^ this could represent a central point for DDR co-ordination directed by RNA-related processes.

Numerous members of the DEAD-box helicase family, including DHX9, DDX1 and Senataxin, comprise yet another growing sub-family of RNA-processing proteins implicated in DNA repair.^11,15,52^ DEAD-box proteins traditionally unwind RNA, although some have been identified to also unwind DNA and DNA*–*RNA hybrids, or R-loops.^63,64^ DDX1 is phosphorylated by ATM and co-localises with γH2AX rapidly in response to irradiation to facilitate HR.^11,65,66^ DHX9 interacts directly with PARP1 and is required for the formation of R-loops.^58,67^ Both DHX9 and Senataxin have been shown to reduce chromosomal instability and promote cell survival.^15,18,58^ Senataxin was suggested to facilitate HR, but not NHEJ, and was found to suppress DSB-induced translocation events.^18^ Importantly, all of these DEAD-box helicases have been shown to directly interact with R-loops, which have recently been shown to be generated around break sites and are currently thought to play a pivotal role in the repair process.^17,18,68–71^

These gene families are not an exhaustive list of RNA-interacting proteins involved in the DDR. On the contrary, several other groups of proteins have also been implicated: exosome complex-associated proteins, including EXOSC10^16,72^ and RBM7,^73^ and intrinsically disordered proteins, such as RBM14^74^ and the FET family (FUS, EWS, TAF15).^75–77^ These intrinsically disordered proteins are characterised by disordered Gly-Arg-Rich (GAR) regions known as RGG boxes.^78^ Despite this similarity, they have a diverse range of biological functions related to RNA processes, and appear to act in a transcription-coupled manner to facilitate DNA repair.^77,79^

### The proteomic scale of RNA-interacting proteins involved in the DDR

**Fig. 2:**
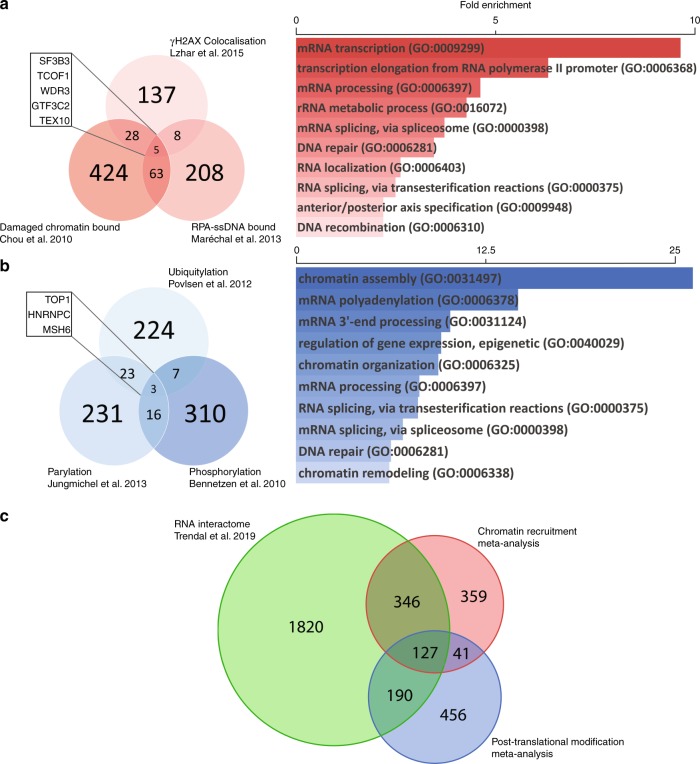
Meta-analysis of proteomic studies into DNA-damage-dependent changes. Venn diagram (left) shows the significant hits shared by each of the studies. Gene ontology slim biological process enrichment analysis (right) shows which gene groups are enriched in the combined list of significant hits from all experiments; the bars represent the fold enrichment of each group. **a** Three studies that investigated proteins that localise/bind to damaged chromatin: association with chromatin in response to UV damage;^80^ co-localisation with γH2AX upon laser micro-irradiation;^77^ and in vitro association with an RPA-ssDNA construct.^81^ **b** Three studies that investigated post-translational modification changes in response to damage: ubiquitylation changes in response to UV damage;^84^ PARylation changes in response to a variety of genotoxic agents;^82^ and phosphorylation changes up to 1 h post 6 Gy of radiation.^85^ **c** Dataset overlap of the combined studies from (**a**) and the combined studies from (**b**) with the RNA interactome.^87^

Numerous studies have used proteomic approaches to identify novel factors involved in DNA damage,^21,22,77,80–86^ often uncovering an abundance of RNA-interacting proteins associated with DNA repair. However, this group of proteins has not been examined in depth, and integrating these studies could uncover novel aspects of the DDR. To gain a wide perspective on the proteins involved in the damage response, we aggregated a number of studies that utilised a variety of different techniques – a general mass-spectrometry approach to identify proteins associated with damaged chromatin,^80^ a screen of tagged proteins for co-localisation with γH2AX in response to UV micro-irradiation^77^ and an invitro-based experiment to investigate interactors of replication protein A (RPA)-coated ssDNA^81^ – each therefore identifying a slightly different subset of DDR factors. Gene ontology analysis on the resulting group of DNA-damage-associated proteins showed that RNA-interacting gene groups were significantly enriched for among the proteins identified, with the top five gene groups all having roles in transcription and transcript processing (Fig. 2a). In parallel, to take a broader view on protein modifications in relation to the DDR, we integrated studies which examined ubiquitylation,^84^ phosphorylation^85^ or polyADP-ribosylation (PARylation)^82^ changes in response to DNA damage. Again, gene ontology analysis identified various RNA-related gene groups as being significantly enriched for (Fig. 2b). RNA-related genes appear to dominate the most enriched groups and have a more significant enrichment than many DNA/chromatin-related groups. Additionally, in a previous investigation of the RNA-binding proteome, it was noted that several DNA repair factors were found to significantly interact with non-poly(A) RNA; 53BP1 was identified as one of the strongest non-poly(A) RNA interactors in the human proteome.^87^ By comparing this RNA interactome with the previously defined groups of chromatin-recruited factors (Fig. 2a) and post-translationally modified (Fig. 2b) proteins, we observed that 54% of proteins recruited to damaged chromatin and 39% of proteins modified in response to damage are RNA-binding proteins (Fig. 2c). This not only reinforces the gene ontology results for these groups, but also highlights RNA-binding as a key feature among canonical DNA repair factors.

This meta-analysis underscores the substantial and fundamental importance of RNA-interacting proteins in the DDR, and suggests that a wide variety of RNA-processing enzymes are not only recruited to damaged chromatin, but are also modified in a number of ways in response to DNA damage, indicating that these are targeted changes directed by the DDR to co-ordinate the repair outcome. These results also suggest that the DDR-interacting, RNA-processing enzymes previously discussed represent only a small fraction of those involved in the pathway, and that the mechanisms of RDDR might be far more complex than currently understood.

### Proposed roles for RNA-interacting enzymes in the DDR

A substantial amount of data clearly implicates a broad group of RNA-processing enzymes in DNA repair. In addition, multiple proteins, including Drosha and WRAP53β, have been shown to control ubiquitin signalling at DSBs, suggesting that they act via a common mechanism; however, our current understanding of the molecular basis of their actions is limited.^17,53,61^ Given that many of these proteins are involved in the regulation of gene expression, it is tempting to postulate that they might regulate the expression of canonical DNA repair factors in response to damage. However, this mode of action is insufficient to explain the rapid responses of these proteins and their localisation to sites of DNA damage, and the lack of a role for other proteins that are involved in the same RNA-processing pathways. For example, the *TNRC6* gene family is required for miRNA-mediated gene silencing, but does not have a role in DNA repair.^13,17^ Instead, it would implicate a mechanism orchestrated by RNA-processing enzymes that acts at a pivotal point in the repair process. There is still a need for further mechanistic insight into how this process works, but R-loops appear to comprise a core component.

## Transcripts are required for the DDR

In light of the significant contributions observed by various RNA-processing enzymes from such a broad range of pathways, it is perhaps not surprising that RNA itself is now also strongly implicated in the repair of DNA damage.^13,17,68–70,88^ Of the enzymes discussed previously, many have been suggested to act via an RNA intermediate at the break site, rather than directly influencing DNA processing/recruitment of repair factors. This then begs the questions: what is the nature of these RNA molecules and what is their role at the break site?

To assess the contribution of cellular RNA to repair factor recruitment, a number of laboratories have used RNase treatment of cells followed by immunofluorescence of repair factors. Treating cells with RNase A as soon as 20 min after irradiation significantly impaired 53BP1 focus formation, while the formation of γH2AX foci was unperturbed. This phenotype can then be reverted by incubation of cells treated with RNase A with nuclear RNA extracted from other cells.^13,89^ This result suggests a direct role for RNA in the recruitment of downstream repair factors, similar to that observed in the studies of various RNA-processing enzymes.^13,17,18,61,65^ At this point, it is possible to propose a mechanism of action for these repair proteins, in which they process an RNA precursor into an active form, DNA-damage response RNA (DDRNA), which then has a role in facilitating efficient repair.^13,14^

Another technique being employed to examine the role of RNA in the response to DNA damage is the use of transcription inhibitors, such as α-amanitin and 5,6-dichlorobenzimidazole 1-β-D-ribofuranoside (DRB), alongside damage induction to observe the effect on repair factor recruitment.^13,68,70,90^ To prevent compensatory alterations in gene expression from confounding these experiments, inhibition of transcription is often done for short periods of time prior to damage induction. Inhibition of transcription reduces the recruitment of NHEJ- and HR-specific factors, suggesting a direct role for transcription in their recruitment.^13,68^ In addition, the reformation of repair foci following the addition of exogenous nuclear RNA described above was shown to be dependent on transcription, implicating nascent transcripts in this mechanism.^13^

## R-Loops: Friend not foe?

**Fig. 3:**
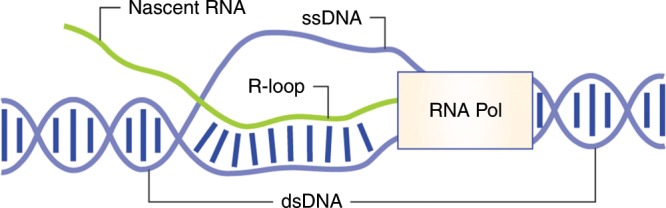
Structure of R-loops and their formation behind RNA polymerases. Partially unannealed double-stranded DNA allows complimentary single-stranded RNA to anneal to one of the free DNA strands. This often occurs behind transcription bubbles, due to the DNA being in an unwound state with the complimentary RNA being actively synthesised and therefore held in close proximity to the DNA.

Although experimental approaches using RNase treatments and transcription inhibitors have proved useful in demonstrating a role for RNA in the DDR, they do not identify the species of RNA involved. Many types of RNA exist within cells, each of which serves different functions and interacts with different cellular components, and therefore the nature of the RNA involved in the repair process will be critical to the mechanism. Given the strong interaction between RNA-processing enzymes and R-loops,^58^ and the fact that DNA*–*RNA hybrids directly connect RNA- and DNA-related processes, the contribution of these RNA species to DNA repair is now being extensively investigated.^17,18,59,71^ The generation of R-loops is tightly linked to active transcription by RNA polymerases, due to the physical proximity of single-stranded DNA to complementary RNA (Fig. 3). Until recently, R-loops were considered to be a source of genome instability due to the increased risk of replication fork collapse, and exposure of the single-stranded non-template DNA to damaging agents.^91–94^ However, R-loops have been shown to not directly cause genome instability^95^ and are now being increasingly implicated in the repair of DNA damage and the preservation of genome stability.^17,68,96^

### Investigating R-loops and R-loop-interacting proteins

RNase H selectively degrades the RNA component of an R-loop and is therefore a powerful tool in the investigation of these structures. Overexpression of RNase H has been shown to significantly reduce both HR and NHEJ efficiency and to delay repair progression via cell-based reporter assays,^17^ while immunofluorescence studies have shown that RNase H overexpression impairs repair factor recruitment in response to DNA damage.^68,71,97,98^ In addition, co-localisation studies using a fluorescently tagged mutant RNase H that binds to, but does not digest, R-loops has shown rapid relocation to sites of DNA damage, suggesting the formation of R-loops.^17,75^ These results indicate that R-loops are generated at break sites to facilitate DDR signal propagation.

The use of RNase H is still a relatively indirect method of investigating R-loops. A more direct and quantitative method is the use of the S9.6 antibody, which has high specificity for DNA*–*RNA hybrids. S9.6 can be used to immunoprecipitate R-loops and has been used in both deep-sequencing (DRIP-Seq) and interactome studies to identify R-loop-binding proteins within the proteome.^17,18,58^ DRIP-Seq studies using inducible DSB cell systems found that R-loops are generated around endogenous DSBs early on during the repair process. Transcription is thought to be shut down in response to DSBs^99–102^ and since canonical R-loops form behind transcription bubbles, in theory, this should reduce the formation of R-loops at break sites. Consistent with this, R-loop levels are reduced across coding regions in response to breaks; however, in close proximity to the breaks, R-loops were generated in response to damage in a manner dependent on transcriptional activity of the locus.^17,18^

The appearance of these R-loops was also found to be dependent on Drosha and their removal dependent on Senataxin, consistent with previous reports identifying DDRNAs as products of Drosha and DICER.^13,17^ These findings suggest that not only is RNA acting directly at the break site, in the form of R-loops, but that RNA-processing enzymes might be acting to facilitate the generation, and possibly also the removal, of these structures in response to damage. This provides invaluable insight into the mechanisms at work here and allows us to begin to paint a picture of how this fascinating pathway functions. Interestingly, an S9.6 interactome study identified both DHX9 and DDX1 as being among the strongest interactors of R-loops along with multiple other RNA-binding proteins that have been implicated in DNA repair.^92,103–105^ Most notable, however, is the identification of DNA-PK and PARP1 as significant R-loop interactors, both of which are core components of the DDR; their knockdown also resulted in significant alterations to global R-loop levels.^58^ This association of R-loops with canonical DNA repair factors not only implicates R-loops further in DNA repair, but also suggests that these structures might contribute to canonical DNA repair processes, rather than simply being a part of a distinct RDDR pathway.

### DNA*–*RNA hybrids and canonical DNA repair

Other canonical DDR factors have also been implicated in processes related to RDDR. RAD52 is required for the assembly of the RAD51 filament around resected ssDNA during HR and is recruited to sites of damage in a manner dependent on transcriptional activity in neuronal cells.^68^ In vitro assays found that RAD52 binds DNA*–*RNA hybrids and single-stranded RNA (ssRNA) as well as double-stranded DNA (dsDNA) and ssDNA. In addition, Mazina et al.^59^ have characterised an additional function of RAD52 in inverse strand exchange between dsDNA and ssRNA. This process allows the formation of a DNA*–*RNA hybrid via the exchange of one of the dsDNA strands with ssRNA and has been shown to facilitate RDDR in yeast cells.^59,68^ In addition, RAD52 was shown to facilitate transcription-associated homologous recombination repair via the processing of R-loops at DSBs.^106^ More recently, BRCA1 and the BRCA1–BARD1 heteroduplex was shown to interact with hybrids, while BRCA2 recruits RNase H2 to facilitate hybrid removal at break sites.^71^ These experiments provide direct evidence of canonical DDR factors not only interacting with R-loops, but also being able to enzymatically utilise RNA in the same way as DNA. Combined with the loss of the recruitment of repair factors upon depletion of RNA-processing enzymes, this suggests that RNA and RNA-processing enzymes co-operate with canonical DNA repair factors to facilitate repair, and that the process of RDDR is strongly linked to canonical DNA repair.

Although some studies on R-loops use RNase-H as a tool for their investigation, most of the data discussed here utilised the S9.6 antibody. This is problematic, as any potential biases from this antibody could skew the results of these experiments. Whereas in vitro experiments found S9.6 to be highly specific towards DNA*–*RNA hybrids,^107^ further investigations have shown that the antibody also exhibits significant affinity towards double-stranded RNA^108^ and variable affinity towards different R-loop sequences.^109^ Off-target binding of S9.6 could therefore be a potential pitfall of these experiments, especially given the complex nucleic acid structures formed during DSB repair.^110^ RNase-H treated samples act as an effective negative control for these experiments; however, these results are not always published. The use of RNase-H in laser micro-irradiation and immunofluorescence experiments does support the formation of R-loops at DSBs; however, much of the data regarding their profile across break sites, protein interactions and dependence on transcriptional activity is dependent on the S9.6 antibody. Thus, although there is convincing evidence for the formation of R-loops at sites of DNA damage, some of these data should be viewed with caution.

## In search of the origin of RNA involved in DNA repair

There are two prevailing theories for the origin of RNA involved in DNA repair: transcription events that occur after break induction and produce RNA from the broken DNA; or the utilisation of a transcript produced prior to break induction that remains in the vicinity of the break. Both theories have extensive supporting evidence, however, identifying the RNA experimentally is challenging as it is difficult to distinguish from background RNA, especially at highly transcribed loci.

### Damage-induced transcription

**Fig. 4:**
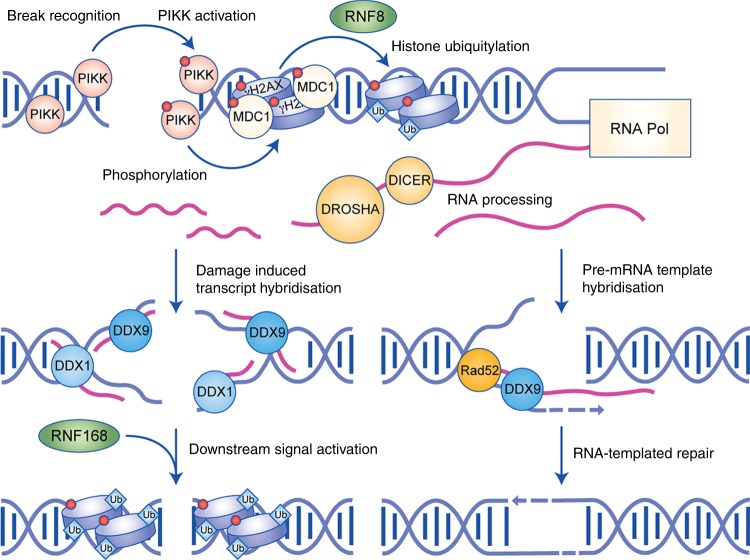
Schematic model of the possible mechanisms of RNA-dependent DNA repair (RDDR). Repair is initiated in the same way as canonical double-stranded break (DSB) repair; however, at the point of the ubiquitylation cascade, an RNA production/processing step occurs, which produces DDRNAs. This DDRNA either takes the form of small RNA, which could hybridise to the broken DNA via the aid of helicases such as DDX1 and DHX9, or of a long RNA molecule, which could bridge the break by hybridising to the DNA via a RAD52-dependent strand invasion mechanism. Either the small RNA acts as a sequence-specific signal and allows propagation of canonical DSB repair pathways, or the long RNA molecule is used as a template for high-fidelity repair.

Upon DSB, the surrounding chromatin gets remodelled via modifications, which causes it to decondense and thereby facilitates the recruitment of repair factors and processing of the DNA. Notably, this chromatin state resembles that of transcriptionally active loci and even harbours some of the same histone modifications.^111^ Until recently, it was thought that all transcription around DSB sites is shut down to prevent transcription over broken DNA thus to avoid collisions/interference of the transcriptional and repair machineries.^99–101,112^ However, it has been hypothesised that this similarity in chromatin conformation between DSB sites and transcriptionally active loci allows the recruitment of RNA polymerase at broken DNA ends and the bi-directional transcription of damage-induced long non-coding RNAs (dilncRNAs).^12,14,69,113–115^ A recent study has shown that, whereas promoter proximal transcription was silenced upon DSB induction, this was then followed by DSB-induced transcription events that occur from the region of the broken DNA ends.^102^ This suggests that dilncRNA transcription at DSBs may be independent of promoter activity, and is instead a distinct mechanism of transcriptional induction regulated by the DDR. It has also been corroborated in multiple organisms that Drosha- and DICER-like enzymes can then digest these dilncRNAs into mature DDRNAs via a mechanism dependent on the transcriptional activity of the damaged locus (Fig. 4).^12,69,115^ These DDRNAs could then be used for targeted degradation of potentially damaged mRNAs, a mechanism that would explain the strong dependence of these small RNAs on RNA-interference enzymes.^12,14,90,113,116^ However, an mRNA silencing mechanism would not explain the dependence of the DDR on these enzymes, since removing these transcripts would not impact the recruitment of repair factors to damage sites or effect repair efficiency. dilncRNAs and DDRNAs have now been shown to form R-loops at break sites,^71,115^ and it has also been suggested that DDRNA can be used as a sequence-specific signal for downstream DDR events (Fig. 4).^69,115,117^ With RNA-processing enzymes commonly functioning in the ubiquitin cascade of the DDR, it could be that DDRNA is not only used as a sequence-specific target for factor recruitment, but also to aid the chromatin remodelling events that occur at this point in the response. R-loops have already been implicated in controlling chromatin conformation, and have even been shown to recruit chromatin-regulatory complexes that are involved in DSB repair.^118–120^ This hypothesis provides a well-rounded explanation for the involvement of RNA in the DDR by linking the canonical roles of implicated enzymes with the RNA-related phenotype. However, the evidence for damage-induced transcription is based on the use of highly expressed reporter systems or break sites flanked by repetitive sequences, which have previously been suggested to confound the results.^12,14,69,114,116^ In addition, RNA sequencing and a variety of next-generation transcription profiling techniques using inducible DSB systems have failed to identify nascent, bi-directional transcription around endogenous break sites in mammalian cells,^14,17,114^ and only recent data have suggested that this scenario might occur at endogenous mammalian loci.^115^ Further research using more endogenous methods is therefore required to support this theory, and to provide a more detailed mechanism for this complex series of events.

### Pre-DSB transcribed nascent mRNA

An alternative theory for the involvement of RNA in the DDR is the possibility that a transcript produced prior to damage induction is processed and incorporated into the repair process in response to DSBs. This has been demonstrated to occur in yeast in an NHEJ-dependent manner using a plasmid reporter, and a recombination-based repair mechanism using RNA was also shown to be dependent on RAD52.^70,88,90^ This theory has gained popularity as a transcript produced prior to the DSB has the potential to maintain the fidelity of the repair by acting as a template for repair.

The possible mechanism of RNA-templated repair is based on enzymes processing transcripts produced prior to break induction to facilitate their hybridisation with the broken DNA, forming an R-loop that can be used as a template.^17,59,68,88,121^ This mechanism would in principle be similar to HR, but importantly could be utilised throughout the cell cycle (Fig. 4). RNA-templated DSB repair has previously been characterised in yeast cells via a mechanism dependent on RAD52 and has been suggested to occur in terminally differentiated mammalian cells.^68,88^ Furthermore, previous research has indicated that transcriptionally active loci are preferentially targeted for templated repair^101^ and, with R-loop generation and the recruitment of RNA-processing enzymes being dependent on transcriptional activity of the loci, it is possible that these phenotypes both occur as a result of the increased levels of potential template RNA in the local environment of highly transcribed genomic regions. RNA-templated repair could therefore provide a direct mechanism to explain how highly transcribed sites have increased rates of repair factor recruitment and templated repair, acting as a safeguarding mechanism for the transcribed, and therefore most active, regions of the genome.

Interestingly, Biehs et al.^122^ found that NHEJ can occur through a resection-dependent mechanism during the G1-phase of the cell cycle that is dependent on a cohort of HR-related factors such as MRE11, CtIP and BRCA1. This was speculated to be an RNA-templated repair mechanism that utilises NHEJ machinery for a rapid response to the break, but in which recruitment of an RNA template initiates a resection-dependent templated repair facilitated by HR-related machinery.^123^ This crossover of factors from both NHEJ and HR is also consistent with the loss of recruitment of factors for both pathways upon knockdown of RNA-processing enzymes.^17,59^ An RNA-templated repair mechanism has also been proposed as the method for high-fidelity DSB repair in terminally differentiated cells, especially considering that cells such as neurons are not rapidly replaced and will therefore accumulate mutations over time.^68,90^

One problem associated with this model, though, is the lack of a mammalian RNA-dependent DNA polymerase other than telomerase; however, there is evidence that DNA replication polymerases, such as polymerases α and δ, can function in this capacity.^88,121^ As it stands, the current data do not provide direct evidence for RNA-templated repair, and this phenomenon is yet to be shown to occur in mammalian cells.^68,70,88^ In addition, it has recently been suggested that R-loops form at DSBs independent of transcriptional activity, instead suggesting damage-induced transcription generates the RNA component of these R-loops.^71^ Significant breakthroughs that demonstrate this mechanism in mammalian cells and that link together the observed phenotypes of G1-phase resection-dependent repair and the seemingly critical role of RAD52 are therefore still required.

### A place for both mechanisms

Both damage-induced transcription and RNA-templated repair provide interesting and logical explanations for the observations made in the discussed studies, and it is, in fact, possible that both mechanisms exist. They might serve separate functions within the DDR, as damaged-induced transcription could provide an early signalling response and a gene-silencing mechanism, whereas RNA-templated repair would act as a downstream pathway similar to NHEJ and HR. However, these are simply two theories for RDDR based on our current knowledge and understanding. Given the importance of RNA in many other processes, it is not unlikely that the role of RNA in the DDR is multifactorial and that RNA acts at multiple points within the pathway to fine-tune the repair process. Although we have focused on DSB repair here, there is also substantial evidence implicating RNA in other branches of DNA repair, such as nucleotide excision repair and the UV response.^86,124,125^ It is likely that there are further roles for RNA in the DDR that currently remain unexplored, especially given the prevalence of RNA-interacting proteins in proteomic studies of the DDR (Fig. 2).

## Implications for cancer research

We have discussed here the evidence that RNA is a vital component of the DDR and that it is required to maintain the fidelity of repair. The issue of how transcriptional landscapes influence DNA repair still needs further investigation, especially given the dynamic nature of the cellular RNA repertoire. This presents many possibilities with regards to how altered cellular conditions, differentiation states and even extracellular signals can influence DNA repair through RNA landscape changes. Moreover, RNA expression is grossly altered in cancer cells.^126,127^ Hyper-transcription leads to highly varied levels of mRNA, miRNA and lncRNA, which vary drastically even between cells from the same cancer subtype due to their heterogeneity.^128^ In addition, cancer cells display extremely high levels of DNA damage from processes such as increased rates of transcription and replication.^129^ Given the substantial role of the DDR in carcinogenesis, further understanding the contribution of RNA to the DDR is essential. Theoretically, mutations in components of RDDR could result in an increased mutation rate, thereby promoting carcinogenesis and cancer progression. Mutations in these repair factors could be linked to altered prognosis, such as reduced survival or resistance to certain therapeutic strategies, and therefore could provide potential prognostic markers aiding our ability to treat patients.^130^

Alternatively, RDDR could act as a critical mechanism that prevents the accumulation of toxic levels of genome instability and mutations in highly expressed genes that are essential for cancer cell survival. Accordingly, further investigation could reveal a host of novel targets for selective therapies that hinder RDDR, resulting in a build-up of mutations at these highly expressed loci that cannot be tolerated by the transformed cells. The possibilities for cancer biology in general are also interesting, as one could imagine an auto-feedback loop of damage and repair at highly expressed gene loci: high levels of transcription would be correlated with higher rates of DSBs, the repair of which can introduce mutations that further increase transcription rates. This feedback loop would be enhanced by mutations in the RDDR machinery and could greatly enhance cancer cell progression. Of course, these are just a few intriguing possibilities and a far deeper understanding of the mechanisms involved here is required to support them.

## Closing remarks

It is becoming clear that RNA contributes to the repair of DSBs via a direct mechanism that is distinct from its role in mediating the expression of DNA repair factors. A broad range of enzymes is required to process RNA in response to DSB induction to facilitate the formation of R-loops at break sites, which are key for the progression of the DDR and probably serve multiple roles. Furthermore, multiple canonical DSB repair factors, including RAD52, PARP1, DNA-PK, BRCA1 and 53BP1, interact with RNA and DNA*–*RNA hybrids.^58,59,131^ The discovery of a repair mechanism that occurs in the G1-phase of the cell cycle and that utilises factors from both HR and NHEJ repair pathways is consistent with phenotypic data for RDDR, such as immunofluorescence^17,61^ and DRIP-seq,^17^ and suggests that RNA is a critical molecule in canonical DSB repair pathways.

When viewing RNA as part of the process of DNA repair, it is common to perceive it as an ‘optional extra’. However, if we consider the possibility of an RNA world,^132,133^ in which life originally evolved from RNA molecules, it becomes almost obvious that RNA would be closely entwined in DNA repair from its conception.^134^ Assuming that DNA evolved from RNA as a stable storage platform for genetic information, RNA could have been required for repair as the original source of genetic information in conjunction with its vital contribution to catalytic processes. Finally, in the burgeoning field of RNA therapeutics^23,24^ it is important to consider how RNA is involved in the repair of DNA. This will allow us to avoid possible issues with RNA therapeutics, such as off-target interactions with DNA repair processes, and, more excitingly, potentially harness the activities of the RDDR in cancer cells to develop novel RNA therapeutic strategies.

## Data Availability

All data presented in this manuscript are open source and have been previously published, and we have cited these publications appropriately.

## References

[1] Ciccia A, Elledge SJ (2010). The DNA damage response: making it safe to play with knives. Mol. Cell.

[2] Richardson C, Jasin M (2000). Frequent chromosomal translocations induced by DNA double-strand breaks. Nature.

[3] Tatsumi-Miyajima J, Yagi T, Takebe H (1993). Analysis of mutations caused by DNA double-strand breaks produced by a restriction enzyme in shuttle vector plasmids propagated in ataxia telangiectasia cells. Mutat. Res./DNA Repair.

[4] Kawanishi S, Ohnishi S, Ma N, Hiraku Y, Oikawa S, Murata M (2017). Nitrative and oxidative DNA damage in infection-related carcinogenesis in relation to cancer stem cells. Genes Environ..

[5] Cao C, Lai T, Li M, Zhou H, Lv D, Deng Z (2016). Smoking-promoted oxidative DNA damage response is highly correlated to lung carcinogenesis. Oncotarget.

[6] Meira LB, Bugni JM, Green SL, Lee CW, Pang B, Borenshtein D (2008). DNA damage induced by chronic inflammation contributes to colon carcinogenesis in mice. J. Clin. Invest.

[7] Memisoglu A, Samson L (2000). Contribution of base excision repair, nucleotide excision repair, and DNA recombination to alkylation resistance of the fission yeast Schizosaccharomyces pombe. J. Bacteriol..

[8] Slyskova J, Sabatella M, Ribeiro-Silva C, Stok C, Theil AF, Vermeulen W (2018). Base and nucleotide excision repair facilitate resolution of platinum drugs-induced transcription blockage. Nucleic Acids Res.

[9] Jackson SP, Bartek J (2009). The DNA-damage response in human biology and disease. Nature.

[10] Mao Z, Bozzella M, Seluanov A, Gorbunova V (2008). Comparison of nonhomologous end joining and homologous recombination in human cells. DNA Repair.

[11] Li L, Monckton EA, Godbout R (2008). A Role for DEAD Box 1 at DNA Double-Strand Breaks. Mol. Cell Biol..

[12] Wei W, Ba Z, Gao M, Wu Y, Ma Y, Amiard S (2012). A Role for Small RNAs in DNA Double-Strand Break Repair. Cell.

[13] Francia S, Michelini F, Saxena A, Tang D, de Hoon M, Anelli V (2012). Site-specific DICER and DROSHA RNA products control the DNA-damage response. Nature.

[14] Michalik K, M, Böttcher R, Förstemann K (2012). A small RNA response at DNA ends in Drosophila. Nucleic Acids Res..

[15] Jain A, Bacolla A, del Mundo IM, Zhao J, Wang G, Vasquez KM (2013). DHX9 helicase is involved in preventing genomic instability induced by alternatively structured DNA in human cells. Nucleic Acids Res.

[16] Marin-Vicente C, Domingo-Prim J, Eberle AB, Visa N (2015). RRP6/EXOSC10 is required for the repair of DNA double-strand breaks by homologous recombination. J. Cell Sci..

[17] Lu WT, Hawley BR, Skalka GL, Baldock RA, Smith EM, Bader AS (2018). Drosha drives the formation of DNA:RNA hybrids around DNA break sites to facilitate DNA repair. Nat. Commun..

[18] Cohen S, Puget N, Lin Y, Clouaire T, Aguirrebengoa M, Rocher V (2018). Senataxin resolves RNA:DNA hybrids forming at DNA double-strand breaks to prevent translocations. Nature. Communications.

[19] Hawley BR, Lu W, Wilczynska A, Bushell M (2017). The emerging role of RNAs in DNA damage repair. Cell Death Differ..

[20] Michelini F, Jalihal AP, Francia S, Meers C, Neeb ZT, Rossiello F (2018). From “Cellular” RNA to “Smart” RNA: Multiple Roles of RNA in Genome Stability and Beyond. Chem. Rev..

[21] Adamson B, Smogorzewska A, Sigoillot FD, King RW, Elledge SJ (2012). A genome-wide homologous recombination screen identifies the RNA-binding protein RBMX as a component of the DNA-damage response. Nat. Cell Biol..

[22] Paulsen RD, Soni DV, Wollman R, Hahn AT, Yee MC, Guan A (2009). A genome-wide siRNA screen reveals diverse cellular processes and pathways that mediate genome stability. Mol. Cell.

[23] Adams D, Suhr OB, Dyck PJ, Litchy WJ, Leahy RG, Chen J (2017). Trial design and rationale for APOLLO, a Phase 3, placebo-controlled study of patisiran in patients with hereditary ATTR amyloidosis with polyneuropathy. BMC Neurol..

[24] Dulla K, Aguila M, Lane A, Jovanovic K, Parfitt DA, Schulkens I (2018). Splice-modulating oligonucleotide QR-110 restores CEP290 mRNA and function in human c.2991+1655A>G LCA10 models. Mol. Ther. Nucleic Acids.

[25] Cao, F., Wan, C., Xie, L., Qi, H., Shen, L., Chen, S. et al. Localized RNA interference therapy to eliminate residual lung cancer after incomplete microwave ablation. *Thorac. Cancer* **10**, 1369–1377 (2019). 10.1111/1759-7714.13079 PMC6558495 31017731

[26] Yin, H., Xiong, G., Guo, S., Xu, C., Xu, R., Guo, P. et al. Delivery of anti-miRNA for triple-negative breast cancer therapy using RNA nanoparticles targeting stem cell marker CD133. *Mol. Ther*. **27**, 1252–1261 (2019). 10.1016/j.ymthe.2019.04.018 PMC6612664 31085078

[27] Berraondo, P., Martini, P. G. V., Avila, M. A. & Fontanellas A. Messenger RNA therapy for rare genetic metabolic diseases. *Gut* **68**, 1323–1330 (2019). 10.1136/gutjnl-2019-318269 30796097

[28] Chen X, Mangala LS, Rodriguez-Aguayo C, Kong X, Lopez-Berestein G, Sood AK (2018). RNA interference-based therapy and its delivery systems. Cancer Metastasis Rev..

[29] Blackford AN, Jackson SP (2017). ATM, ATR, and DNA-PK: the Trinity at the Heart of the DNA Damage Response. Mol. Cell.

[30] Finzel A, Grybowski A, Strasen J, Cristiano E, Loewer A (2016). Hyperactivation of ATM upon DNA-PKcs inhibition modulates p53 dynamics and cell fate in response to DNA damage. Mol. Biol. Cell.

[31] Sirbu, B. M. & Cortez, D. DNA damage response: three levels of DNA repair regulation. *Cold Spring Harb. Perspect. Biol.* **5**, a012724, (2013). 10.1101/cshperspect.a012724 PMC3721278 23813586

[32] Lips J, Kaina B (2001). DNA double-strand breaks trigger apoptosis in p53-deficient fibroblasts. Carcin.

[33] van den BergJ, G, Manjón A, Kielbassa K, Feringa FM, Freire R, Medema R (2018). A limited number of double-strand DNA breaks is sufficient to delay cell cycle progression. nar.

[34] Schwertman P, Bekker-Jensen S, Mailand N (2016). Regulation of DNA double-strand break repair by ubiquitin and ubiquitin-like modifiers. Nat. Rev. Mol. Cell Biol..

[35] Thorslund T, Ripplinger A, Hoffmann S, Wild T, Uckelmann M, Villumsen B (2015). Histone H1 couples initiation and amplification of ubiquitin signalling after DNA damage. Nature.

[36] Mattiroli F, Vissers JA, van Dijk W, Ikpa P, Citterio E, Vermeulen W (2012). RNF168 Ubiquitinates K13-15 on H2A/H2AX to Drive DNA Damage Signaling. Cell.

[37] Shibata A (2017). Regulation of repair pathway choice at two-ended DNA double-strand breaks. Mutat. Res.

[38] Isono M, Niimi A, Oike T, Hagiwara Y, Sato H, Sekine R (2017). BRCA1 directs the repair pathway to homologous recombination by promoting 53BP1 dephosphorylation. Cell Rep..

[39] Escribano-Diaz C, Orthwein A, Fradet-Turcotte A, Xing M, Young JT, Tkac J (2013). A cell cycle-dependent regulatory circuit composed of 53BP1-RIF1 and BRCA1-CtIP controls DNA repair pathway choice. Mol. Cell.

[40] Reid DA, Keegan S, Leo-Macias A, Watanabe G, Strande NT, Chang HH (2015). Organization and dynamics of the nonhomologous end-joining machinery during DNA double-strand break repair. Proc. Natl Acad. Sci. USA.

[41] Wang YG, Nnakwe C, Lane WS, Modesti M, Frank KM (2004). Phosphorylation and regulation of DNA ligase IV stability by DNA-dependent protein kinase. J. Biol. Chem..

[42] Cottarel J, Frit P, Bombarde O, Salles B, Négrel A, Bernard S (2013). A noncatalytic function of the ligation complex during nonhomologous end joining. J. Cell Biol..

[43] Zhou Y, Caron P, Legube G, Paull TT (2014). Quantitation of DNA double-strand break resection intermediates in human cells. Nucleic Acids Res.

[44] Miki Y, Swensen J, Shattuck-Eidens D, Futreal PA, Harshman K, Tavtigian S (1994). A strong candidate for the breast and ovarian cancer susceptibility gene BRCA1. Science.

[45] Claus EB, Schildkraut J, Iversen ES, Berry D, Parmigiani G (1998). Effect of BRCA1 and BRCA2 on the association between breast cancer risk and family history. J. Natl. Cancer Inst..

[46] Sandoval N, Platzer M, Rosenthal A, Dork T, Bendix R, Skawran B (1999). Characterization of ATM gene mutations in 66 ataxia telangiectasia families. Hum. Mol. Genet.

[47] Lewis KA, Bakkum-Gamez J, Loewen R, French AJ, Thibodeau SN, Cliby WA (2007). Mutations in the ataxia telangiectasia and rad3-related-checkpoint kinase 1 DNA damage response axis in colon cancers. Genes Chromosomes Cancer.

[48] Boeckman HJ, Trego KS, Turchi JJ (2005). Cisplatin sensitizes cancer cells to ionizing radiation via inhibition of nonhomologous end joining. Mol. Cancer Res..

[49] Wan B, Dai L, Wang L, Zhang Y, Huang H, Qian G (2018). Knockdown of BRCA2 enhances cisplatin and cisplatin-induced autophagy in ovarian cancer cells. Endocr. Relat. Cancer.

[50] Fong PC, Boss DS, Yap TA, Tutt A, Wu P, Mergui-Roelvink M (2009). Inhibition of poly(ADP-ribose) polymerase in tumors from BRCA mutation carriers. N. Engl. J. Med..

[51] Bryant HE, Schultz N, Thomas HD, Parker KM, Flower D, Lopez E (2005). Specific killing of BRCA2-deficient tumours with inhibitors of poly(ADP-ribose) polymerase. Nature.

[52] Becherel OJ, Yeo AJ, Stellati A, Heng EYH, Luff J, Suraweera AM (2013). Senataxin plays an essential role with DNA damage response protein meiotic recombination and gene silencing. PLOS Genet..

[53] Pederiva C, Böhm S, Julner A, Farnebo M (2016). Splicing controls the ubiquitin response during DNA double-strand break repair. Cell Death Differ..

[54] Francia S, Cabrini M, Matti V, Oldani A, d’Adda di Fagagna F (2016). DICER, DROSHA and DNA damage response RNAs are necessary for the secondary recruitment of DNA damage response factors. J. Cell Sci..

[55] Krietsch J, Caron M, Gagné J, Ethier C, Vignard J, Vincent M (2012). PARP activation regulates the RNA-binding protein NONO in the DNA damage response to DNA double-strand breaks. Nucleic Acids Res..

[56] Beli P, Lukashchuk N, Wagner SA, Weinert BT, Olsen JV, Baskcomb L (2012). Proteomic investigations reveal a role for RNA processing factor THRAP3 in the DNA damage response. Mol. Cell.

[57] Ribeiro de Almeida C, Dhir S, Dhir A, Moghaddam AE, Sattentau Q, Meinhart A (2018). RNA helicase DDX1 converts RNA G-Quadruplex structures into R-loops to promote IgH class switch recombination. Mol. Cell.

[58] Cristini A, Groh M, Kristiansen MS, Gromak N (2018). RNA/DNA hybrid interactome identifies DXH9 as a molecular player in transcriptional termination and R-loop-associated DNA damage. Cell Rep..

[59] Mazina OM, Keskin H, Hanamshet K, Storici F, Mazin AV (2017). Rad52 inverse strand exchange drives RNA-templated DNA double-strand break repair. Mol. Cell.

[60] Coucoravas C, Dhanjal S, Henriksson S, Böhm S, Farnebo M (2017). Phosphorylation of the Cajal body protein WRAP53β by ATM promotes its involvement in the DNA damage response. RNA Biol..

[61] Henriksson S, Rassoolzadeh H, Hedstrom E, Coucoravas C, Julner A, Goldstein M (2014). The scaffold protein WRAP53beta orchestrates the ubiquitin response critical for DNA double-strand break repair. Genes Dev..

[62] Mandemaker IK, van Cuijk L, Janssens RC, Lans H, Bezstarosti K, Hoeijmakers JH (2017). DNA damage-induced histone H1 ubiquitylation is mediated by HUWE1 and stimulates the RNF8-RNF168 pathway. Sci. Rep..

[63] Martin-Tumasz S, Brow DA (2015). Saccharomyces cerevisiae Sen1 helicase domain exhibits 5’- to 3’-helicase activity with a preference for translocation on DNA rather than RNA. J. Biol. Chem..

[64] Song C, Hotz-Wagenblatt A, Voit R, Grummt I (2017). SIRT7 and the DEAD-box helicase DDX21 cooperate to resolve genomic R loops and safeguard genome stability. Genes Dev..

[65] Li L, Germain DR, Poon H, Hildebrandt MR, Monckton EA, McDonald D (2016). DEAD Box 1 facilitates removal of RNA and homologous recombination at DNA double-strand breaks. Mol. Cell Biol..

[66] Li L, Poon HY, Hildebrandt MR, Monckton EA, Germain DR, Fahlman RP (2017). Role for RIF1-interacting partner DDX1 in BLM recruitment to DNA double-strand breaks. DNA Repair (Amst.).

[67] Chakraborty P, Huang JTJ, Hiom K (2018). DHX9 helicase promotes R-loop formation in cells with impaired RNA splicing. Nat. Commun..

[68] Welty S, Teng Y, Liang Z, Zhao W, Sanders LH, Greenamyre JT (2018). RAD52 is required for RNA-templated recombination repair in post-mitotic neurons. J. Biol. Chem..

[69] Michelini F, Pitchiaya S, Vitelli V, Sharma S, Gioia U, Pessina F (2017). Damage-induced lncRNAs control the DNA damage response through interaction with DDRNAs at individual double-strand breaks. Nat. Cell Biol..

[70] Chakraborty A, Tapryal N, Venkova T, Horikoshi N, Pandita RK, Sarker AH (2016). Classical non-homologous end-joining pathway utilizes nascent RNA for error-free double-strand break repair of transcribed genes. Nat. Commun..

[71] D’Alessandro G, Whelan DR, Howard SM, Vitelli V, Renaudin X, Adamowicz M (2018). BRCA2 controls DNA:RNA hybrid level at DSBs by mediating RNase H2 recruitment. Nat. Commun..

[72] Domingo-Prim J, Endara-Coll M, Bonath F, Jimeno S, Prados-Carvajal R, Friedländer MR (2019). EXOSC10 is required for RPA assembly and controlled DNA end resection at DNA double-strand breaks. Nat. Commun..

[73] Blasius M, Wagner SA, Choudhary C, Bartek J, Jackson SP (2014). A quantitative 14-3-3 interaction screen connects the nuclear exosome targeting complex to the DNA damage response. Genes Dev..

[74] Simon NE, Yuan M, Kai M (2017). RNA-binding protein RBM14 regulates dissociation and association of non-homologous end joining proteins. Cell Cycle.

[75] Britton S, Dernoncourt E, Delteil C, Froment C, Schiltz O, Salles B (2014). DNA damage triggers SAF-A and RNA biogenesis factors exclusion from chromatin coupled to R-loops removal. Nucleic Acids Res..

[76] Rulten SL, Rotheray A, Green RL, Grundy GJ, Moore DAQ, Gómez-Herreros F (2013). PARP-1 dependent recruitment of the amyotrophic lateral sclerosis-associated protein FUS/TLS to sites of oxidative DNA damage. nar.

[77] Izhar L, Adamson B, Ciccia A, Lewis J, Pontano-Vaites L, Leng Y (2015). A systematic analysis of factors localized to damaged chromatin reveals PARP-dependent recruitment of transcription factors. Cell Rep..

[78] Ozdilek BA, Thompson VF, Ahmed NS, White CI, Batey RT, Schwartz JC (2017). Intrinsically disordered RGG/RG domains mediate degenerate specificity in RNA binding. nar.

[79] Hill SJ, Mordes DA, Cameron LA, Neuberg DS, Landini S, Eggan K (2016). Two familial ALS proteins function in prevention/repair of transcription-associated DNA damage. Proc. Natl Acad. Sci. USA.

[80] Chou DM, Adamson B, Dephoure NE, Tan X, Nottke AC, Hurov KE (2010). A chromatin localization screen reveals poly (ADP ribose)-regulated recruitment of the repressive polycomb and NuRD complexes to sites of DNA damage. Proc. Natl. Acad. Sci. USA.

[81] Maréchal A, Li JM, Ji XY, Wu CS, Yazinski SA, Nguyen HD (2014). PRP19 transforms into a sensor of RPA-ssDNA after DNA damage and drives ATR activation via a ubiquitin-mediated circuitry. Mol. Cell.

[82] Jungmichel S, Rosenthal F, Altmeyer M, Lukas J, Hottiger MO, Nielsen ML (2013). Proteome-wide identification of poly(ADP-Ribosyl)ation targets in different genotoxic stress responses. Mol. Cell.

[83] Matsuoka S, Ballif BA, Smogorzewska A, McDonald ER, Hurov KE, Luo J (2007). ATM and ATR substrate analysis reveals extensive protein networks responsive to DNA damage. Science.

[84] Povlsen LK, Beli P, Wagner SA, Poulsen SL, Sylvestersen KB, Poulsen JW (2012). Systems-wide analysis of ubiquitylation dynamics reveals a key role for PAF15 ubiquitylation in DNA-damage bypass. Nat. Cell Biol..

[85] Bennetzen MV, Larsen DH, Bunkenborg J, Bartek J, Lukas J, Andersen JS (2010). Site-specific Phosphorylation Dynamics of the Nuclear Proteome during the DNA Damage Response. Mol. Cell Proteom..

[86] Boeing S, Williamson L, Encheva V, Gori I, Saunders RE, Instrell R (2016). Multiomic analysis of the UV-induced DNA damage response. Cell Rep..

[87] Trendel J, Schwarzl T, Horos R, Prakash A, Bateman A, Hentze MW (2019). The human RNA-binding proteome and its dynamics during translational arrest. Cell.

[88] Keskin H, Shen Y, Huang F, Patel M, Yang T, Ashley K (2014). Transcript-RNA-templated DNA recombination and repair. Nature.

[89] Pryde F, Khalili S, Robertson K, Selfridge J, Ritchie A, Melton DW (2005). 53BP1 exchanges slowly at the sites of DNA damage and appears to require RNA for its association with chromatin. J. Cell Sci..

[90] Wei L, Nakajima S, Böhm S, Bernstein KA, Shen Z, Tsang M (2015). DNA damage during the G0/G1 phase triggers RNA-templated, Cockayne syndrome B-dependent homologous recombination. Proc. Natl Acad. Sci. USA.

[91] Amon, J. D. & Koshland D. RNase H enables efficient repair of R-loop induced DNA damage. *elife* https://doi.org/10.7554/eLife.20533 (2016) 10.7554/eLife.20533 PMC5215079 27938663

[92] Gan W, Guan Z, Liu J, Gui T, Shen K, Manley JL (2011). R-loop-mediated genomic instability is caused by impairment of replication fork progression. Genes Dev..

[93] Sorrells, S., Nik, S., Casey, M., Cameron, R. C., Truong, H., Toruno, C., et al. Spliceosomal components protect embryonic neurons from R-loop-mediated DNA damage and apoptosis. *Dis. Model Mech*. https://doi.org/10.1242/dmm.031583 (2018). 10.1242/dmm.031583 PMC5894942 29419415

[94] Yang Z, Hou Q, Cheng L, Xu W, Hong Y, Li S (2017). RNase H1 cooperates with DNA gyrases to restrict R-loops and maintain genome integrity in Arabidopsis Chloroplasts. Plant Cell.

[95] García-Pichardo D, Cañas JC, García-Rubio ML, Gómez-González B, Rondón AG, Aguilera A (2017). Histone mutants separate R loop formation from genome instability induction. Mol. Cell.

[96] Ohle C, Tesorero R, Schermann G, Dobrev N, Sinning I, Fischer T (2016). Transient RNA-DNA hybrids are required for efficient double-strand break repair. Cell.

[97] Garcia-Rubio ML, Perez-Calero C, Barroso SI, Tumini E, Herrera-Moyano E, Rosado IV (2015). The fanconi anemia pathway protects genome integrity from R-loops. PLoS Genet.

[98] Tumini E, Barroso S, Calero CP, Aguilera A (2016). Roles of human POLD1 and POLD3 in genome stability. Sci. Rep..

[99] Shanbhag NM, Rafalska-Metcalf IU, Balane-Bolivar C, Janicki SM, Greenberg RA (2010). An ATM-dependent transcriptional silencing program is transmitted through chromatin in cis to DNA double strand breaks. Cell.

[100] Pankotai T, Bonhomme C, Chen D, Soutoglou E (2012). DNAPKcs-dependent arrest of RNA polymerase II transcription in the presence of DNA breaks. Nat. Struct. Mol. Biol..

[101] Marnef A, Cohen S, Legube G (2017). Transcription-coupled DNA double-strand break repair: active genes need special care. J. Mol. Biol..

[102] Vítor AC, Sridhara SC, Sabino JC, Afonso AI, Grosso AR, Martin RM (2019). Single-molecule imaging of transcription at damaged chromatin. Sci. Adv..

[103] Chen L, Luo C, Shen L, Liu Y, Wang Q, Zhang C (2017). SRSF1 prevents DNA damage and promotes tumorigenesis through regulation of DBF4B pre-mRNA splicing. Cell Rep..

[104] Taira N, Yamaguchi T, Kimura J, Lu Z, Fukuda S, Higashiyama S (2014). Induction of amphiregulin by p53 promotes apoptosis via control of microRNA biogenesis in response to DNA damage. Proc. Natl Acad. Sci. USA.

[105] Morales JC, Richard P, Patidar PL, Motea EA, Dang TT, Manley JL (2016). XRN2 links transcription termination to DNA damage and replication stress. PLoS Genet.

[106] Yasuhara T, Kato R, Hagiwara Y, Shiotani B, Yamauchi M, Nakada S (2018). Human Rad52 promotes XPG-mediated R-loop processing to initiate transcription-associated homologous recombination repair. Cell.

[107] Phillips DD, Garboczi DN, Singh K, Hu Z, Leppla SH, Leysath CE (2013). The sub-nanomolar binding of DNA-RNA hybrids by the single-chain Fv fragment of antibody S9.6. J. Mol. Recognit..

[108] Hartono SR, Malapert A, Legros P, Bernard P, Chédin F, Vanoosthuyse V (2018). The affinity of the S9.6 antibody for double-stranded RNAs impacts the accurate mapping of R-loops in Fission Yeast. J. Mol. Biol..

[109] Konig F, Schubert T, Langst G (2017). The monoclonal S9.6 antibody exhibits highly variable binding affinities towards different R-loop sequences. PLoS One.

[110] Bzymek M, Thayer NH, Oh SD, Kleckner N, Hunter N (2010). Double Holliday junctions are intermediates of DNA break repair. Nature.

[111] Ziv Y, Bielopolski D, Galanty Y, Lukas C, Taya Y, Schultz DC (2006). Chromatin relaxation in response to DNA double-strand breaks is modulated by a novel ATM- and KAP-1 dependent pathway. Nat. Cell Biol..

[112] Shanbhag NM, Rafalska-Metcalf IU, Balane-Bolivar C, Janicki SM, Greenberg RA (2010). ATM-dependent chromatin changes silence transcription in cis to DNA double-strand breaks. Cell.

[113] Lee H, Chang S, Choudhary S, Aalto AP, Maiti M, Bamford DH (2009). qiRNA is a new type of small interfering RNA induced by DNA damage. Nature.

[114] Bonath F, Domingo-Prim J, Tarbier M, Friedlander MR, Visa N (2018). Next-generation sequencing reveals two populations of damage-induced small RNAs at endogenous DNA double-strand breaks. Nucleic Acids Res..

[115] Burger, K., Schlackow, M., Gullerova, M. Tyrosine kinase c-Abl couples RNA polymerase II transcription to DNA double-strand breaks. *Nucleic Acids Res.* **47**, 3467–3484 (2019). 10.1093/nar/gkz024 PMC6468493 30668775

[116] Miki D, Zhu P, Zhang W, Mao Y, Feng Z, Huang H (2017). Efficient generation of diRNAs requires components in the posttranscriptional gene silencing pathway. Sci. Rep..

[117] Gao M, Wei W, Li M, Wu Y, Ba Z, Jin K (2014). Ago2 facilitates Rad51 recruitment and DNA double-strand break repair by homologous recombination. Cell Res.

[118] Chen PB, Chen HV, Acharya D, Rando OJ, Fazzio TG (2015). R loops regulate promoter-proximal chromatin architecture and cellular differentiation.. Nat. Struct. Mol. Biol..

[119] Campbell S, Ismail IH, Young LC, Poirier GG, Hendzel MJ (2013). Polycomb repressive complex 2 contributes to DNA double-strand break repair. Cell Cycle.

[120] Xu Y, Sun Y, Jiang X, Ayrapetov MK, Moskwa P, Yang S (2010). The p400 ATPase regulates nucleosome stability and chromatin ubiquitination during DNA repair. J. Cell Biol..

[121] Storici F, Bebenek K, Kunkel TA, Gordenin DA, Resnick MA (2007). RNA-templated DNA repair. Nature.

[122] Biehs R, Steinlage M, Barton O, Juhasz S, Kunzel J, Spies J (2017). DNA double-strand break resection occurs during non-homologous end joining in G1 but is distinct from resection during homologous recombination. Mol. Cell.

[123] Löbrich M, Jeggo P (2017). A process of resection-dependent nonhomologous end joining involving the Goddess Artemis. Trends Biochem. Sci..

[124] Chitale S, Richly H (2018). DICER- and MMSET-catalyzed H4K20me2 recruits the nucleotide excision repair factor XPA to DNA damage sites. J. Cell Biol..

[125] Li CL, Golebiowski FM, Onishi Y, Samara NL, Sugasawa K, Yang W (2015). Tripartite DNA lesion recognition and verification by XPC, TFIIH, and XPA in nucleotide excision repair. Mol. Cell.

[126] Schwarzer A, Emmrich S, Schmidt F, Beck D, Ng M, Reimer C (2017). The non-coding RNA landscape of human hematopoiesis and leukemia. Nat. Commun..

[127] Tang XH, Urvalek AM, Osei-Sarfo K, Zhang T, Scognamiglio T, Gudas LJ (2015). Gene expression profiling signatures for the diagnosis and prevention of oral cavity carcinogenesis-genome-wide analysis using RNA-seq technology. Oncotarget.

[128] Lee W, Diao L, Wang J, Zhang J, Roarty EB, Varghese S (2018). Multiregion gene expression profiling reveals heterogeneity in molecular subtypes and immunotherapy response signatures in lung cancer. Mod. Pathol..

[129] Tubbs A, Nussenzweig A, Endogenous DNA (2017). Damage as a source of genomic instability in cancer. Cell.

[130] Zhu Y, Wu J, Zhang C, Sun S, Zhang J, Liu W (2016). BRCA mutations and survival in breast cancer: an updated systematic review and meta-analysis. Oncotarget.

[131] Castello A, Horos R, Strein C, Fischer B, Eichelbaum K, Steinmetz LM (2016). Comprehensive identification of RNA-binding proteins by RNA interactome capture. Methods Mol. Biol..

[132] Gilbert W (1986). Origin of life: the RNA world. Nature.

[133] Pressman A, Blanco C, Chen IA (2015). The RNA world as a model system to study the origin of life. Curr. Biol..

[134] Robertson MP, Joyce GF (2012). The Origins of the RNA world. Cold Spring Harb. Perspect. Biol..

